# Specificity and Affinity Quantification of Flexible Recognition from Underlying Energy Landscape Topography

**DOI:** 10.1371/journal.pcbi.1003782

**Published:** 2014-08-21

**Authors:** Xiakun Chu, Jin Wang

**Affiliations:** 1 College of Physics, Jilin University, Changchun, Jilin, P. R. China; 2 State Key Laboratory of Electroanalytical Chemistry, Changchun Institute of Applied Chemistry, Chinese Academy of Sciences, Changchun, Jilin, P. R. China; 3 Department of Chemistry and Physics, State University of New York at Stony Brook, Stony Brook, New York, United States of America; UNC Charlotte, United States of America

## Abstract

Flexibility in biomolecular recognition is essential and critical for many cellular activities. Flexible recognition often leads to moderate affinity but high specificity, in contradiction with the conventional wisdom that high affinity and high specificity are coupled. Furthermore, quantitative understanding of the role of flexibility in biomolecular recognition is still challenging. Here, we meet the challenge by quantifying the intrinsic biomolecular recognition energy landscapes with and without flexibility through the underlying density of states. We quantified the thermodynamic intrinsic specificity by the topography of the intrinsic binding energy landscape and the kinetic specificity by association rate. We found that the thermodynamic and kinetic specificity are strongly correlated. Furthermore, we found that flexibility decreases binding affinity on one hand, but increases binding specificity on the other hand, and the decreasing or increasing proportion of affinity and specificity are strongly correlated with the degree of flexibility. This shows more (less) flexibility leads to weaker (stronger) coupling between affinity and specificity. Our work provides a theoretical foundation and quantitative explanation of the previous qualitative studies on the relationship among flexibility, affinity and specificity. In addition, we found that the folding energy landscapes are more funneled with binding, indicating that binding helps folding during the recognition. Finally, we demonstrated that the whole binding-folding energy landscapes can be integrated by the rigid binding and isolated folding energy landscapes under weak flexibility. Our results provide a novel way to quantify the affinity and specificity in flexible biomolecular recognition.

## Author Summary

Flexibility in biomolecular recognition is crucial for the function. Flexibility often leads to moderate binding affinity but high binding specificity, challenging the conventional wisdom that high specificity is guaranteed by high affinity. Currently, understanding of the relationship between affinity and specificity in flexible biomolecular recognition is still obscure, even in a qualitative way. By exploring the intrinsic biomolecular recognition energy landscapes, we provided a novel way to quantify the thermodynamic intrinsic specificity by energy landscape topography and kinetic specificity by association rate. We show quantitatively that flexibility decreases binding affinity while increases binding specificity, and the relative changes in affinity and specificity are strongly correlated with the degree of flexibility. Our results show that more (less) flexibility leads to weaker (stronger) coupling between affinity and specificity. Importantly, we demonstrated that flexibility modulates affinity and specificity through the underlying energy landscape. Our study establishes the quantitative relationship among flexibility, affinity and specificity, bridging the gap between theory and experiments.

## Introduction

The key for a cell surviving and functioning is through specific biomolecular recognition, which is controlled by non-covalent interactions, such as van der Waals forces, electrostatic forces, hydrogen bonds and hydrophobic forces [1], [2]. Understanding the process of biomolecular recognition is crucial for biology [3]–[5] and also for the development of rational drug discovery [6]–[9]. To describe biomolecular recognition, there are two essential ingredients: binding affinity and binding specificity. The affinity, which is defined as the free energy differences between native binding and unbound states, measures the stability of native binding states [10]. While the conventional specificity is defined as the affinity of binding to one relative to the other targets. From the definitions, we can see that affinity and specificity are coupled. In drug discovery, highly efficient and specific pharmacological activity of a drug requires both affinity and specificity. However in many cellular activities, such as cellular signaling and regulatory process, high affinity is often absent. It is due to the fact that the strong interactions in the complex would make the duration of binding too long, leading to low efficient function. In particular, highly specific biomolecular recognition 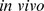 is often associated with conformational changes or folding, and proceeds with fast association and dissociation rate, resulting in low affinity [11]–[21]. Evidence has been accumulating that flexibility in biomolecular recognition is critical for realizing biomolecular function with high specificity [12], [22]–[45]. This challenges the long-standing paradigm that the function is determined by the unique structure. Therefore, flexibility seems to on one hand decrease affinity, and on the other hand increase specificity. This decoupling between affinity and specificity has brought out a new issue that the concept of binding specificity in flexible recognition may need to be redefined without consideration of binding affinity.

To quantify the conventional binding specificity, one has to explore all the possible binding targets and then calculate the discriminations among affinities (relative affinity). However, it is often impractical in reality. From our energy landscape theory, the definition of the conventional specificity, which measures the discriminations of binding to different targets, can be transformed to the intrinsic specificity, which measures the discriminations of binding to a single target with different binding modes, under the assumption that the targeted proteins are big enough [46]–[49]. Intrinsic specificity describes the distributions of binding modes in the associated complex states and therefore is determined by the topography of the binding energy landscapes [50]–[52]. In practice, the steeper slope to the native binding states, the smoother energy landscape surface and the smaller configurational searching space of the energy funnel, leads to more specific binding complex and then more intrinsic specific binding [46], [47]. Now it is recognized that the intrinsic specificity is correlated with the conventional specificity [49], so quantification of binding specificity is feasible in practice through quantifying the topography of the binding energy landscape instead of exploring all the binding targets. In addition, the efficiency of biomolecular function 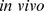 can be evaluated by the fast association rate [53]. To quantify the binding specificity in flexible biomolecular recognition, both the thermodynamic and kinetic aspects should be included. Although there are many qualitative analyses on how flexibility in biomolecular recognition decreases binding affinity and increases binding specificity [37], [54]–[56], the quantitative results remain unexplored in both simulations and experiments. In our previous investigation [57], we have demonstrated that both the thermodynamics and kinetics of flexible biomolecular recognition are determined by the topography of the intrinsic energy landscapes. Consequently, to quantify binding affinity and specificity in flexible recognition, as well as the role of flexibility in uncoupling of affinity and specificity, we need to explore the intrinsic binding energy landscape.

It has been recognized that binding can facilitate folding in flexible biomolecular recognition. Recently, increasing evidence that some proteins can only fold upon binding to their targets have put forward a new folding scheme, called "binding induced folding" [12], [22], [24], [25], [31], [33], [38], [44], [58]–[61]. For this "intrinsically disordered proteins" (IDPs), the folding thermodynamic stability and kinetic behavior are quite different in isolated and dimeric environment. Since the folding thermodynamics and kinetics are determined by the topography of the underlying energy landscapes [62], it is undoubted that the folding energy landscapes of IDPs have been changed by binding during the recognition process. In order to investigate the role of interfacial binding in folding, we have to quantify the intrinsic folding energy landscapes. In our previous studies [57], we have quantified the individual flexible interfacial binding and monomeric folding induced by binding, as well as the whole global energy landscapes in binding-folding dynamics. These folding and binding energy landscapes are strongly coupled and are regarded as "effective energy landscapes". It has been suggested that the whole energy landscapes can be combined by the binding and folding energy landscapes if the binding and folding are weakly coupled in the recognition process [63]. These individual binding and folding energy landscapes describe how the interfacial binding proceeds without much consideration of monomeric folding, in accordance with rigid docking, and how the monomeric folding proceeds without much consideration of interfacial binding, in accordance with isolated folding. These uncoupled binding and folding energy landscapes are therefore regarded as "independent energy landscapes". In weak coupling of binding and folding case, the whole binding-folding landscape topography quantities, such as energy gap, energy roughness and entropy can be combined from these three individual independent energy landscapes (two for folding and one for binding) and this whole binding-folding energy landscape is therefore regarded as "combined energy landscape".

By now, it is still unclear how to quantitatively investigate the role of flexibility in biomolecular recognition, especially in binding affinity and specificity, as well as the role of binding in the process of induced folding. In the following work, we meet the challenge by quantifying the rigid and flexible binding, folding with and without binding, the combined and whole global energy landscapes for five 3-state (non-obligatory) homodimers using density of states. We proposed that binding specificity can be described by the intrinsic binding energy landscape topography measure 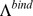 in thermodynamics and association rate in kinetics. The thermodynamic and kinetic specificity are found to be strongly correlated. From the quantified binding energy landscapes, we found that flexibility decreases the binding affinity but increases the thermodynamic and kinetic binding specificity. Furthermore, the proportions of decreasing affinity and increasing specificity are found to be strongly correlated with the increasing flexibility. We have concluded that the conventional specificity can be mapped to the intrinsic specificity under large protein limit and these two specificities are found to be correlated [49]. The results here showed that more (less) flexibility leads to weaker (stronger) coupling between affinity and specificity in biomolecular recognition, consistent with the previous qualitative thought-experiments [54], [55]. Therefore, we established the quantitative relationship among flexibility, affinity and specificity. From quantified folding energy landscapes, we found that folding with binding slightly increases folding stability and shows a slightly more funneled folding energy landscape. By comparing the combined energy landscapes with the whole global energy landscapes, we found that the discriminations of the topography between them are small and strongly correlated with interfacial flexibility. It demonstrated that the whole binding-folding energy landscapes can be deduced from the individual binding and folding energy landscapes with weak binding and folding coupling, as predicated by the theory [63]. Our methods quantitatively investigated the importance of flexibility in binding affinity and specificity, as well as the role of binding in induced folding in biomolecular recognition, therefore provide a novel way to bridge the gap between theoretical analyses and experimental measurements.

## Results

### Quantifying the binding affinity

**Figure 1:**
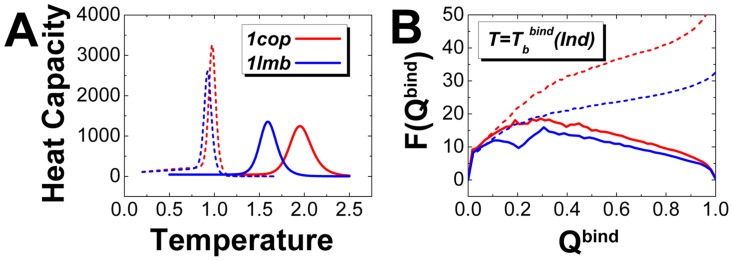
The binding affinity (stability) for rigid (independent) and flexible (effective) binding shown in (A) heat capacity curves and (B) free energy landscapes for Lambda Cro repressor (PDB: 1cop) and Lambda repressor (PDB: 1lmb). The solid and corresponding dotted lines represent rigid and flexible binding respectively. Free energy landscapes are plotted at the rigid binding transition temperatures, which are calculated from the peaks of heat capacity curves for binding, respectively. Free energy is in reduced unit. 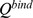 is the fraction of native interfacial binding contacts. "Ind" and "Eff" are the abbreviations for "Independent" and "Effective" binding, respectively.

In order to see how flexibility participates in biomolecular recognition, we investigated the rigid and flexible binding of five 3-state homodimers using structure based model. To be consistent, we used the same interfacial binding interactions, namely the same interfacial contact map for rigid and flexible binding. The heat capacity curves (Figure 1A and Figure S2A in Text S1) clearly show that rigid binding increases the binding transition temperatures, which are obtained by the peaks of the heat capacity curves, compared with flexible binding. Besides, the heat capacity curves of rigid binding show wider peak distributions than flexible binding, implying the binding transition are less cooperative when the monomers are frozen. By investigating the free energy landscapes (Figure 1B and Figure S2B, S2C in Text S1) for rigid and flexible binding both at the binding transition temperatures, we can see that rigid binding has a more stable native binding state with deeper free energy minima biased to 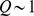, compared with flexible binding. The heat capacity curves and free energy landscapes have led to the fact that freezing the monomers increases binding stability but decreases binding cooperativity. Therefore, we can conclude that flexibility has significantly reduced binding affinity during the recognition process, consistent with the previous experimental investigations [12], [24], [31], [33], [38], [44], [59].

**Figure 2:**
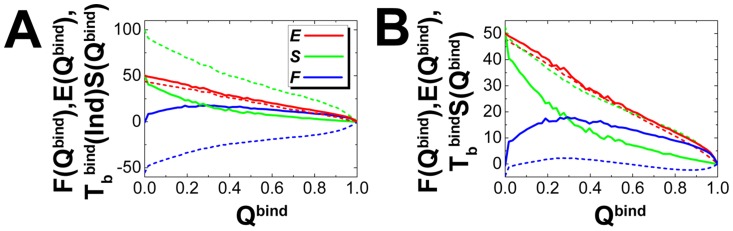
The energy, entropy and free energy of rigid and flexible binding for Lambda Cro repressor. The profiles are plotted at (A) rigid binding temperature (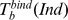), and (B) corresponding rigid and flexible binding transition temperature (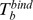). Energy, entropy and free energy are in reduced unit.

The free energy changes between the unbound and native binding states, i.e. the binding affinity, composed of enthalpic and entropic terms, determine the feasibility of biomolecular recognition under certain environment. Here we investigated the details of the components in free energy for rigid and flexible binding at the binding transition temperatures (Figure 2). We find that the energetic terms almost remain the same in both rigid and flexible binding, while the entropic terms show significant deviations in the two cases and are therefore the major elements to control the tendency of the free energy. At unbound states, there are more states for searching in flexible than rigid binding at the rigid binding transition temperature (Figure 2A), resulting in larger contributions of entropic term to lower the binding affinity. Furthermore, at the corresponding rigid and flexible binding transition temperatures (Figure 2B), we can see that the free energy has a lower barrier height in flexible than rigid binding, implying a faster association with flexibility at binding transition temperature. The differences in free energy profiles are due to the increasing entropy at the binding transition states with flexibility. In conclusions, we claim that flexibility or dynamics resulting in significant increasing entropic contributions dominate the free energy and therefore controls the binding thermodynamics and kinetics [64].

### Quantifying the binding specificity in thermodynamics and kinetics

In our drug design strategy, the intrinsic specificity, which is the topography measure of binding energy landscapes, is used for quantifying the binding specificity [46]–[48]. Intrinsic specificity is found to be correlated with conventional specificity and can be a practical substitute for conventional specificity, of which the procedure of evaluating is complicated [49]. Similarly, we adapted the concept of intrinsic specificity from drug discovery to flexible biomolecular recognition. To calculate the intrinsic specificity, we need to quantify the binding energy landscape. By calculating the density of states, we quantified the rigid and flexible binding energy landscapes and projected them onto the energy and configuration space for visualization (Figure S3 in Text S1). We can see that the rigid binding energy landscapes significantly reduce the number of states, i.e. the space of the configuration entropy, without changing the energy scale, leading to smaller-sized and similar deep funnels, compared with the flexible binding energy landscapes.

**Figure 3:**
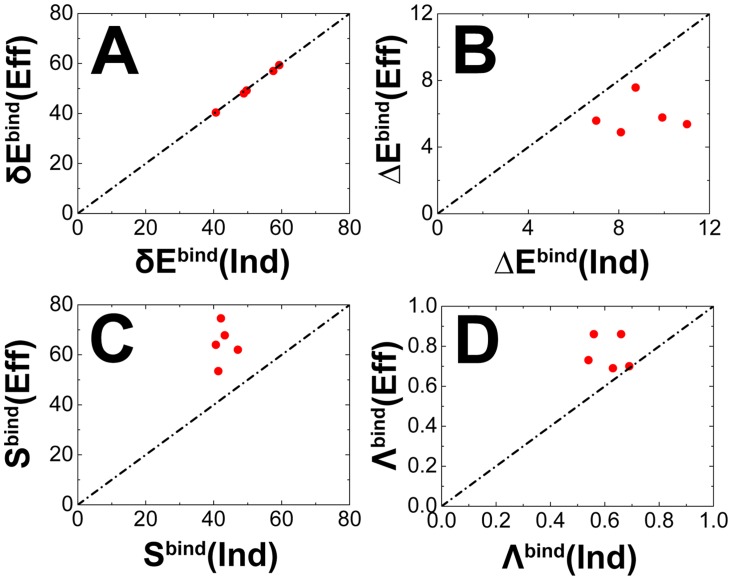
The relationship of the topography of energy landscapes between rigid and flexible binding. The quantities of topography of energy landscapes are shown in (A) energy gap 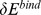, (B) energy roughness 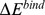, (C) entropy 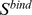 and (D) energy landscape topography measure 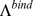.

By quantifying the energy landscape using density of states [57], [62], we calculated the energy gap 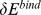, energy roughness 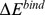 and entropy 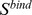 for rigid and flexible binding energy landscapes. From Table 1, we can see that the energy gaps between native states and average of non-native states are similar, due to the identical inter-chain interactions (Figure 3A). Meanwhile, the rigid binding energy landscapes have smaller entropy of non-native states and larger energy roughness than flexible binding energy landscapes (Figure 3B and 3C). In other words, the conformational flexibility, on one hand increases the sizes of the energy landscapes to become less funneled, and on the other hand smoothes the surface of the energy landscapes to be more funneled. In practice, the two effects are opposite for the topography of the energy landscapes and can be captured by the dimensionless ratio 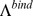 as the ratio of the gap against roughness modularized by the entropy (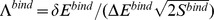). 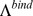 is the topography measure of binding energy landscapes and is used for quantifying the intrinsic binding specificity.

**Table 1:** Quantified rigid and flexible binding energy landscapes.

PDB	1cop		1lmb		1lfb		3mus		1xso	
	Ind a	Eff	Ind	Eff	Ind	Eff	Ind	Eff	Ind	Eff
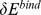	57.56	57.02	48.89	48.03	49.70	49.19	40.63	40.41	59.31	59.35
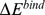	11.01	5.37	9.92	5.77	8.09	4.89	7.00	5.58	8.74	7.57
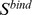	42.12	74.58	40.67	64.00	43.27	67.79	41.36	53.47	47.11	62.03
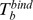	1.95	0.97	1.59	0.92	1.62	0.98	1.39	0.97	1.66	1.21
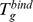	1.20	0.44	1.10	0.51	0.87	0.42	0.77	0.54	0.90	0.68
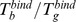	1.62	2.20	1.45	1.80	1.86	2.33	1.80	1.79	1.84	1.78
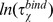	9.92	7.08	9.70	7.64	9.44	7.50	7.92	7.08	7.64	9.21
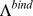	0.56	0.86	0.54	0.73	0.66	0.86	0.63	0.69	0.69	0.70
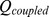	0.18		0.17		0.16		0.12		0.09	

Energy is in reduced unit; Temperature is in energy unit.

a “Ind” and “Eff” are the abbreviations for “Independent” and “Effective” binding, respectively.

From Table 1, we can see that adding conformational flexibility in binding leads to increasing 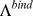 (Figure 3D), corresponding to more funneled binding energy landscapes and therefore more specific binding. Our results are consistent with the qualitative analyses that flexibility in biomolecular recognition increases binding specificity [54], [55]. Notice that the intrinsic specificity 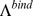 of superoxide dismutase (PDB: 1xso) are very similar for rigid and flexible binding. It is due to the fact that the interfacial flexibility in superoxide dismutase is so small that flexibility is supposed to have little effect on the topography of the binding energy landscape.

**Figure 4:**
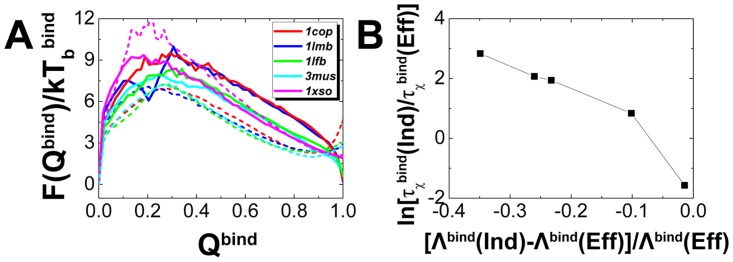
The differences of thermodynamics and kinetics between rigid and flexible binding. (A) The free energy landscapes of rigid and flexible binding are plotted at the corresponding binding temperature 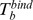. (B) The differences of kinetics, represented by the ratio of binding time between rigid and flexible binding, are plotted along the differences of intrinsic specificity between rigid and flexible binding.

The efficiency of biomolecular function is controlled by association and dissociation rate 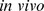. The fact that fast kinetic rate leads to highly efficient function, provides us a way to quantify the specificity through kinetic approach as association rate [53]. In previous studies, flexibility was found to accelerate biomolecular recognition by "fly-casting" mechanism [23], [65]–[68]. Here, we performed a series of kinetic simulations for each homodimer with and without flexibility and calculated the corresponding association rates. We found that flexibility facilitates the binding, except superoxide dismutase. The kinetic results in Table 1 are consistent with the free energy landscapes (Figure 4A and Figure S4A in Text S1), where the binding barrier heights are lowered when flexibility is involved. It implies that the association and dissociation rates are accelerated by flexibility, leading to highly efficient function in flexible recognition. Increasing association rate induced by flexibility leads to increasing kinetic specificity, consistent with the previous qualitative results [11]–[21]. It is worth noting that there is a counter-example in superoxide dismutase, of which rigid binding is faster than flexible binding. In fact, the relationships between the differences of the on- and off- rates, as well as free energy in rigid and flexible binding are not always straightforward. In association, if folding is the rate-limiting step, increasing flexibility will increase the free energy barrier and thus slow down the binding kinetics. On the other hands, if folding is not the rate-limiting, increasing flexibility will decrease the free energy barrier and thus accelerate the binding kinetics by "fly-casting" effect. For superoxide dismutase, the association rate is found to decrease with flexibility, implying that flexibility decreases kinetic specificity. The abnormal relationship between flexibility and kinetic specificity is due to the fact that there is little interfacial flexibility in superoxide dismutase to facilitate binding, so folding of the monomers becomes the rate-limiting step for the association.

In order to have a comparison between intrinsic and kinetic specificity, we investigated the association rates with topography measures of quantified energy landscapes. We can see a strong monotonous increasing relationship between association rate and energy landscape topography measure in rigid binding (Figure S4B in Text S1). The result is consistent with our previous conclusions that the topography of the energy landscapes determines the protein folding and binding kinetics [57], [62]. In addition, we found that the decreasing ratio of association time is monotonously correlated with the increasing proportion of 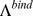 (Figure 4B), indicating that the changes in quantified energy landscapes topography, induced by flexibility, controls the changes in binding kinetics. Therefore, we established a strong quantitative correlation between intrinsic and kinetic specificity. It is worth noting that superoxide dismutase has very similar intrinsic specificity but quite different kinetic specificity in rigid and flexible binding, implying that when there is little interfacial flexibility, the intrinsic specificity, lacking of explicit consideration of the monomeric folding, will have a deviation from the kinetic specificity, which is quantified by the association rate including both binding and folding. Therefore, we argue that the intrinsic and kinetic specificity are strongly correlated when there is adequate flexibility at the interfacial surfaces of the associated biomolecules. In reality, the efficiency of biomolecular recognition should take account of both intrinsic and kinetic specificity.

### Flexibility modulates affinity and specificity through the intrinsic energy landscapes

**Figure 5:**
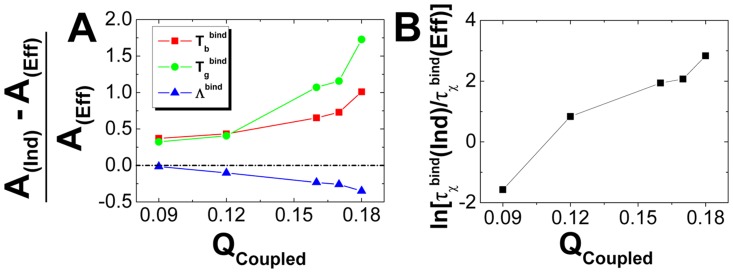
The differences between rigid and flexible binding energy landscapes changes with interfacial flexibility. (A) The differences between rigid and flexible binding energy landscapes are described by the 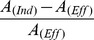, where 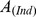 and 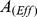 are the quantities of the rigid and flexible binding. The quantities 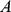 are 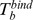, 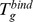 and 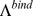, corresponding to the binding transition temperature, glassy trapping temperature and binding energy landscape topography measure, respectively. (B) The differences of kinetics, represented by the ratio of association time between rigid and flexible binding, are plotted as a function of 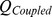. 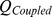 describes the degree of interfacial flexibility.

To see how flexibility influences the binding affinity and specificity, we investigated the differences of affinity and specificity between rigid and flexible binding. Flexibility is quantified by 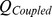, which is the fraction of the residues forming the contacts both in inter- and intra-chains interactions in native structure. 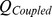 is also the criterion to measure the interfacial folding flexibility with consideration of binding and can be regarded as the coupled degree of the binding and folding. As we can see from Figure 5A, the differences in 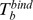, 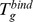 between the rigid and flexible binding are monotonously increasing with 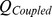, implying that increasing flexibility will decrease the binding transition temperatures and glassy trapping temperatures. Notice that the binding temperature 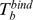 reflects binding stability and can be used to measure binding affinity. Therefore the decreasing binding affinity is strongly correlated with the increasing degree of flexibility with different homodimers. By investigating the intrinsic and kinetic specificity, we found that the differences in 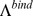 and association time between rigid and flexible binding increase as 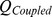 increases. This indicates that increasing flexibility correlates with increasing binding specificity both in thermodynamics and kinetics. We found that the conventional specificity is correlated with the intrinsic specificity [49]. The results here showed that more (less) flexibility leads to weaker (stronger) coupling between affinity and specificity. This is the quantitative relationship among flexibility, affinity and specificity. In our previous analysis, 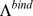 as the topography measure of energy landscapes, is found to be the underlying factor to control the thermodynamics and kinetics of binding. Consequently, we argue that flexibility modulates the thermodynamics and kinetics by changing the topography of the energy landscape. In conclusions, flexibility can be used as a way to decrease binding affinity and increase binding specificity to achieve recognition benefits. Since 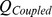, 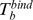, 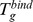 and the association time can be explicitly measured [69]–[71], our results can be directly linked to the experiments.

### Flexibility modulates the folding energy landscapes in recognition

**Figure 6:**
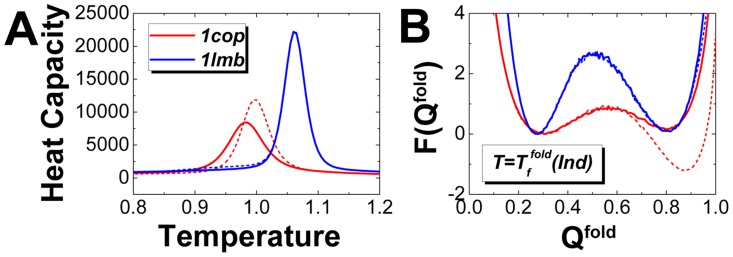
The folding stability for folding with and without interfacial binding shown in (A) heat capacity curves and (B) free energy landscapes for Lambda Cro repressor (red) and Lambda repressor (blue). The solid and corresponding dotted lines represent isolated (independent) and dimeric (effective) folding respectively. Free energy landscapes are plotted at the isolated folding transition temperatures, which are calculated from the peaks of heat capacity curves for folding, respectively. Free energy is in reduced unit.

In order to see how interfacial binding influences the monomeric folding in biomolecular recognition, we investigated folding of isolated monomers and folding of monomers in dimeric environment using structure based model. To be consistent, we used the same monomeric folding interactions, namely the same monomeric contact map in isolated and dimeric folding. We show that the folding heat capacity curves change a little with and without interfacial binding (Figure 6A and Figure S6A in Text S1). For all the homodimers in our work except Lambda repressor, the dimeric environment enhance the monomeric folding stability and cooperativity, with heat capacity curves representing a slightly higher folding temperature and a slightly shaper peak for folding transition than isolated folding. The results can also be deduced from the free energy landscapes at the folding temperatures with and without binding (Figure 6B, Figure S6B and S6C in Text S1). Compared with isolated folding, folding with binding slightly biases the free energy towards the folding basin, resulting in a more stable folding states. In addition, we can see that although the folding stability is enhanced, the folding barrier height is not influenced. In other words, the folding transition state does not change with and without interfacial binding, indicating that the monomeric folding enhancement by the interfacial binding happens at the late stage of the folding. Intriguingly, we also found that for Lambda repressor, there is no differences between the folding in isolated and dimeric environment. It is due to the fact that for this homodimer, binding happens between two completely folded units rather than involving unfolding units, so the binding has no influence on folding and is not expected to change the thermodynamic stability for the monomeric folding.

By calculating the density of states, we quantified the folding energy landscapes with and without interfacial binding (Figure S7 in Text S1). We found that these two energy landscapes are all very similar, implying that interfacial binding does not influence the folding energy landscapes significantly for these investigated 3-state homodimers. By quantifying the energy landscape quantities using density of states (Table 2), we also found that the topography of the energy landscapes are similar between folding with and without binding. It is expected, since the 3-state homodimers can fold the isolated monomers into ordered structures alone without the partners. Although the dimeric environment is able to smooth the funneled energy landscape by decreasing the energy roughness, and finally results in a more funneled energy landscape with slightly increasing 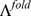, the differences of the energy landscape topography measure 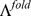 between folding with and without binding are in 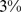. Therefore, we conclude that the interfacial binding interactions help rather than determine the monomers to fold for 3-state homodimers, and serve to fine-tune rather than reshape the topography of the funneled folding energy landscapes.

**Table 2:** Quantified folding energy landscapes with and without interfacial binding.

PDB	1cop		1lmb		1lfb		3mus		1xso	
	Ind a	Eff	Ind	Eff	Ind	Eff	Ind	Eff	Ind	Eff
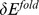	205.95	205.75	303.79	304.44	248.46	246.06	285.31	288.39	626.12	628.95
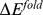	9.34	9.06	11.44	11.44	10.12	9.72	10.88	10.87	17.41	17.29
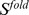	189.35	185.92	261.70	261.57	222.12	223.36	257.11	256.32	501.29	494.16
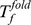	0.98	1.00	1.06	1.06	0.99	1.00	1.02	1.02	1.22	1.24
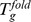	0.48	0.47	0.50	0.50	0.48	0.46	0.48	0.48	0.55	0.55
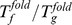	2.04	2.13	2.12	2.12	2.06	2.17	2.12	2.12	2.22	2.25
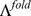	1.13	1.17	1.16	1.16	1.16	1.19	1.15	1.17	1.13	1.15

Because the homodimer is formed by two identical chains, the folding properties of the two monomers for each homodimer are expected to be same.

a “Ind” and “Eff” are the abbreviations for “Independent” and “Effective” folding, respectively.

### Flexibility modulates the whole binding-folding energy landscapes

**Figure 7:**
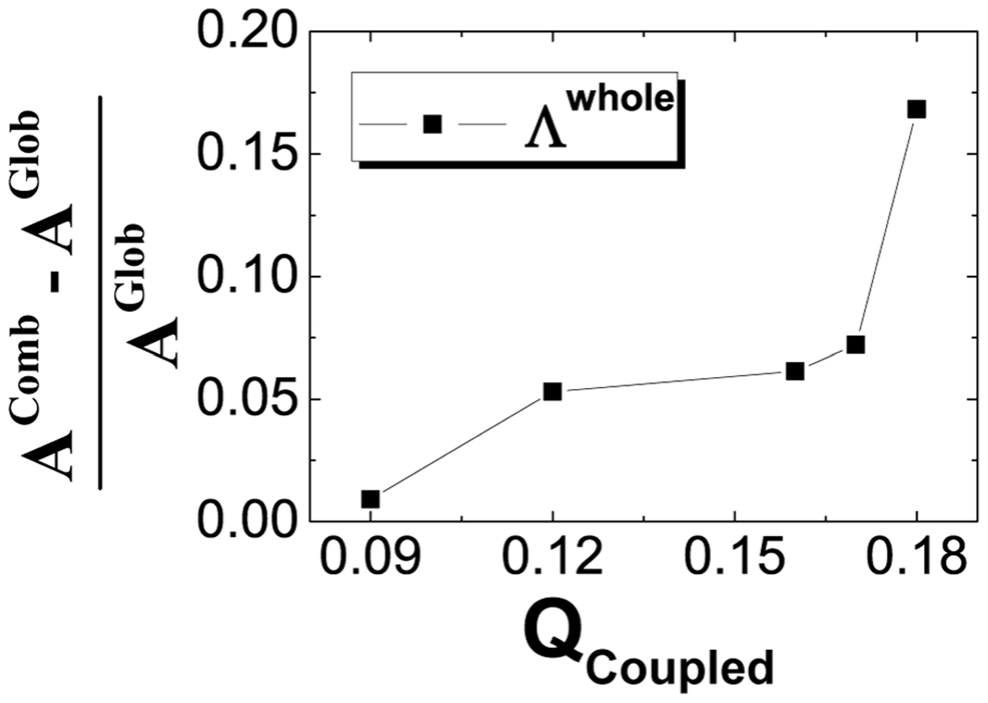
The evolution of the differences between the combined and whole global binding-folding energy landscapes as a function of 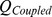. The difference between the combined and whole global binding-folding energy landscapes is described by the 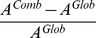, where 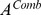 and 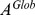 are the energy landscape topography measures 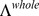 of the combined and whole global binding-folding. "Comb" and "Glob" are the abbreviations for "combined" and "whole global" binding-folding, respectively.

In our previous theoretical investigation, the whole binding-folding energy landscape can be constructed from a combination of one interfacial binding and two monomeric folding energy landscapes under weak coupling of binding and folding [63]. This whole binding-folding energy landscape can be regarded as the combined binding-folding energy landscape and its topography can be quantitatively calculated from the three weakly coupled interfacial binding and monomeric folding energy landscapes, corresponding to rigid binding and isolated folding energy landscapes, respectively. In our previous work [57], the whole binding-folding energy landscapes have also been quantified by directly calculating the density of states from the binding-folding dynamics without the weak coupling assumption and are referred as the whole "global" binding-folding energy landscapes. Here we compared these two whole binding-folding energy landscapes with and without the weakly coupling assumptions. We found that the combined energy landscapes show larger energy gaps, entropies and energy roughness than the whole global energy landscapes (Table S2 in Text S1), implying that the funnels of the combined energy landscapes are deeper, larger and smoother than that of the whole global energy landscapes. By quantifying the energy landscape topography measure 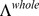, the whole global energy landscapes represented more funneled topography than the combined energy landscapes with larger value of 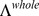. It indicates that the whole global energy landscapes, optimizing the delicate balance of the inter- and intra-chain interactions to decrease the energy roughness and decrease the entropy by coupled binding and folding, will lead to more funneled energy landscapes. Furthermore, we also found that the differences of the energy landscape topography between the two whole energy landscapes are monotonously correlated with the 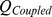 (Figure 7), which is used for describing the interfacial flexibility or the coupled degree of binding and folding in flexible biomolecular recognition. This shows as the binding and folding couples stronger to each other (more flexibility), the whole binding-folding energy landscape is less accurately described by the individual independent binding and folding. Here, for the five 3-state homodimers, the topography of the two landscapes are similar, with the differences of energy landscape topography measure 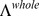 all in 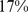, our simulation results are consistent with the theoretical predictions [63]. Therefore our findings provide a novel way of using intrinsic energy landscape approach to investigate flexible biomolecular recognition through individual binding and folding dynamics for certain weak binding-folding coupling proteins.

## Discussion

There are two related but distinct aspects to describe biomolecular recognition: affinity and specificity. Binding affinity is easy to measure in experiments, while quantifying binding specificity is complicated. It has been demonstrated that the conventional binding specificity is correlated with the intrinsic binding specificity [49], which is the measure of the intrinsic binding energy landscape topography [47]–[49]. The argument provides us a way to quantify the binding specificity through the energy landscape approach. In reality, specificity should ensure both thermodynamic feasibility and kinetic efficiency, which is often measured by the kinetic rate. Since the free energy landscape in canonical ensemble, which controls affinity and kinetic rate, can be deduced from the underlying intrinsic energy landscape, which is a description of density of states in micro-canonical ensemble by: 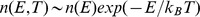, both affinity and specificity can be quantified by the intrinsic energy landscape topography, which are less dependent on the environment and are therefore the reflections of the underlying intrinsic interactions of the system.

It has been recognized that flexibility or folding in biomolecular recognition decreases binding affinity and increases binding specificity [54], [55], [72], [73]. The intrinsic disordered characteristic has benefitted IDPs' binding for fast association and dissociation rate with high specific biomolecular function when IDPs are participating into the function of the living cells [12], [23], [56], [67], [74]–[81]. In general, binding with flexibility, leading to certain amount of disorder in unbound states, will result in a large contribution of entropy term into free energy in unbound states, thus will decrease binding affinity [82]. On the other hand, binding with a certain degree of disorder can allow a larger contact surface with abundant interfacial interactions, compared to the stable proteins [83]–[85], it will lead to an increasing specificity [24]–[26], [59], of which the interactions are mainly governed by the structural complementarity, of the binding partners. This qualitative understanding of flexibility or folding participating into binding with moderate affinity and high specificity is now widespread. However, this uncoupling between affinity and specificity in flexible recognition challenges the traditional viewpoint that high specificity is often accompanied with high affinity. Flexible recognition with moderate affinity but high specificity has brought up a new possibility that the concept of specificity may be redefined without consideration of affinity and the quantitative analysis of the uncoupled affinity and specificity in flexible biomolecular recognition needs to be established. In our studies, we meet the challenge by quantifying the intrinsic energy landscapes.

Using density of states, we quantified the binding and folding energy landscapes for five 3-state homodimers in biomolecular recognition. By investigating rigid and flexible binding, we found that binding affinity decreases with flexibility through the heat capacity curves and free energy profiles. The decreasing proportion of binding affinity is found to be strongly correlated with the increasing flexibility for different homodimers. In addition, by investigating the free energy profiles, we found that the changes of the free energy landscapes in rigid and flexible binding are mainly contributed by the entropic term with flexible binding showing a larger number of states to explore, while the energy term remains similar in the two cases. The quantitative results are consistent with the qualitative understanding that the disorder-to-order transition is always associated with a conformational entropic cost, resulting in a decreasing binding affinity [37], [86]–[88]. It is worth noting that the binding cooperativity, which is obtained from the heat capacity curves, is found to be enhanced by flexibility. Although entropy is increased by flexibility, energy roughness is decreased, simultaneously. Finally the two combined effects lead to the increasing binding cooperativity enhanced by flexibility. Therefore, increasing flexibility can be regarded as a practical way to improve the binding cooperativity in biomolecular recognition. To determine binding specificity, we quantified the binding energy landscape topography measure 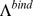 as the thermodynamic intrinsic specificity. Compared with the rigid binding energy landscapes, the flexible binding energy landscapes are characterized by larger sized, similar slope and smoother funnels, corresponding to larger entropy, similar energy gap and smaller energy roughness, resulting in larger values of 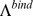. Larger 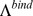 corresponds to more funneled energy landscapes, leading to higher intrinsic specificity. Furthermore, the increasing intrinsic specificity is found to be strongly correlated with the degree of flexibility. In summary, we established the quantitative relationship among flexibility, affinity and specificity in thermodynamics for biomolecular recognition.

As the efficiency of biomolecules realizing their function in experiment measurements is mostly dependent on kinetics rather than thermodynamics, binding specificity can be defined in a kinetic way by using association rate or residence time. In current drug design, the residence time [89]–[94], which is the duration of the drug-target complex, is already used to determine the drug's efficiency. The longer time the drug resides on the receptor, the longer biological effect endures. With similar binding affinity, the residence time can be quite different and therefore can be an indicator to discriminate the different receptors. In our drug design strategy, we evaluated the kinetic specificity by using residence time. We found that the conventional, kinetic and intrinsic specificity are found to be correlated in drug-target binding [49]. In flexible recognition, high efficiency of the biomolecular function is guaranteed by fast association/dissociation and similar affinity may correspond to various association/dissociation rates. Therefore, kinetic rate can be an indicator to discriminate different partners. Similarly, we evaluate the kinetic specificity by using association rate in flexible recognition.

It is well-known that IDPs realize their function with high association and dissociation rates in regulatory and signaling. The fast on-rate, leading to high kinetic specificity, is a result of decreasing the free energy barrier with flexibility by "fly-casting" mechanism [23], [65]–[68]. In practice, we found that whether the association rate increases with flexibility depends on which one of folding or binding is the rate-limiting step in flexible biomolecular recognition. The kinetic results reflect the function of the investigated homodimers 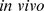. In details, the first three dimers (PDB: 1cop, 1lmb, 1lfb) (Figure S1 in Text S1) are found to participate into the process of gene regulation [95]–[97], in which flexibility can lead to fast association/dissociation rates and thus highly efficient function. While superoxide dismutase (PDB: 1xso) is ubiquitous metalloenzyme that catalyzes the dismutation of the toxic superoxide radical into oxygen and hydrogen peroxide [98], there is little flexibility in the monomers of superoxide dismutase and the kinetic rate is decelerated by flexibility, leading to a stable structural scaffold for functions. The quantitative relationship between flexibility and binding rate is established in our studies as increasing flexibility is strongly correlated with the changes of association rate. This is also the quantitative relationship between flexibility and kinetic specificity with increasing flexibility corresponding to increasing kinetic specificity. In our previous results [57], [62], we have found a strong monotonous correlation between the energy landscape topography and folding/binding rates. Here we conform that the topography of the intrinsic energy landscape reshaped by flexibility or folding is still the underlying factor to determine the changes of association rates, namely the kinetic specificity in flexible biomolecular recognition with respect to rigid docking. Therefore, we established the strong correlation between the kinetic and intrinsic specificity. In reality, the kinetic specificity can be explicitly measured in experiments while the intrinsic specificity, which is a theoretical prediction, can be calculated by the topography of the energy landscapes using density of states. The strong correlated relationship between kinetic specificity and intrinsic specificity in flexible recognition, provides a novel way to link the theoretical predictions to the experiment measurements. Furthermore, from experimental measurements, the underlying landscape topography for binding can be inferred.

From free energy landscapes, we found that the increasing association rate in flexible recognition is mainly due to the fact that flexibility or folding decreases the free energy barrier height by increasing entropy. Entropy is expected to have dramatically opposite roles in kinetic specificity and intrinsic specificity, since entropy positively and negatively correlates with association rate and 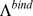, respectively. From quantified energy landscapes, we found that flexibility on one hand increases entropy, and on the other hand decreases energy roughness. Indeed, biomolecules in flexible recognition seem to optimize the interactions through adapting the disordered structures to achieve a smoother energy landscapes with sacrifice of increasing the entropy. Finally the combined two effects both increase association rate and 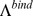 in flexible binding with respect to rigid binding. It is worth noting that the interfacial contacts in our work for rigid and flexible binding are kept the same in the setup. This implies that the contact surfaces in bound states in the two cases are the same, corresponding to the same energy gap, which is confirmed in our studies. The same interfacial contacts here lead to the fact that the proportions of the binding specificity that derived from the interfacial structural complementarity are the same in rigid and flexible binding. Therefore, the increasing specificity in flexible binding with respect to rigid binding in our studies is due to the fact that flexibility or folding modulates the process of recognition by increasing entropy loss and decreasing energy roughness. This leads to the increasing kinetic efficiency (entropy loss increases) and thermodynamic feasibility (roughness decreases), simultaneously. It is worth noting that in the previous qualitative discussions of flexibility and specificity [37], [42], [54]–[56], interfacial contacts are always assumed to be more through flexible structural complementarity, compared to rigid binding. For our case, where the interface is kept the same in flexible and rigid binding, the previous analyses would conclude that the specificity of flexible recognition and rigid docking are the same. Here we point out that the specificity of flexible recognition is higher than the rigid docking even when the interfacial contacts are the same, due to the lowering thermodynamic barrier from larger entropy loss and the same energy gap but smoother underlying energy landscapes. We can take into account of the contact surface changes due to flexibility or folding in the previous specificity studies. The binding contact surface strengths are expected to be quantitatively correlated with the energy gap monotonously. When flexibility is introduced in the system, energy gap is increased, specificity is also expected to be increased, consistent with the previous studies. Our new quantification of specificity states that specificity is not only dependent on energy gap correlated with the number and the strengths of the interfacial contacts, but also dependent on entropy loss and underlying energy landscape roughness. Furthermore, our new quantification provides the link between the specificity and the underlying binding/recognition energy landscape topography (gap, roughness and size measured by the entropy), where previous studies have been mostly focused on the link between the specificity and the interfacial interactions. Therefore, our new definition of specificity is a generation to the previous one. Overall, we argue that in reality, binding specificity in biomolecular recognition is controlled by both entropic term (entropy) in kinetics and energetic term (energy roughness and energy gap) in thermodynamics. These three quantities are directly related to the topography of the binding energy landscapes. Therefore, our results indicate that flexibility modulates the thermodynamics and kinetics through the underlying energy landscapes, therefore is critical for biomolecular recognition.

By investigating folding with and without interfacial binding, we found that the folding thermodynamic stability increases a little and the folding energy landscape becomes slightly more funneled in dimeric environment. However, the topography of the isolated folding energy landscapes are similar with the folding energy landscapes in dimeric environment, representing similar energy gap, roughness, entropy and finally similar 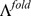. It indicates that the monomers of 3-state homodimers can fold into the ordered structures no matter whether the other chain exists or not. The funneled energy landscapes are not supposed to change significantly with and without interfacial binding. In other words, we can conclude that interfacial binding helps rather than determines folding for our investigated proteins.

Finally, we combined the rigid binding and isolated folding intrinsic energy landscapes into the combined binding-folding energy landscapes according to the theory [63]. We found that the topography of the combined energy landscapes are similar to that of the whole global energy landscapes, which are directly deduced from the binding-folding dynamics [57], consistent with the theory [63]. However, since the independent binding and folding energy landscapes are completely decoupled, the combined energy landscapes are a little different with the whole global energy landscapes, which are comprised of coupled effective binding and folding energy landscapes, represented by increasing energy gap, energy roughness and entropy. The topography of energy landscape ratio 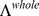 of the combined energy funnels are found to be a little smaller than the whole global energy funnels, and the differences are monotonously correlated with the degree of flexibility and the coupled degree of inter-chain and intra-chain interactions for different proteins. Therefore, in weak coupling of binding and folding (less flexibility), we can combine the whole binding-folding landscape by individual binding and folding. While for strongly coupled binding and folding (more flexibility), the whole global coupled landscapes, instead of individual binding and folding landscapes are required.

In conclusion, we quantified the topography of intrinsic binding and folding energy landscapes, as well as the combined and the whole global energy landscapes. Using the quantified energy landscapes, we quantitatively investigated the role of flexibility in binding thermodynamics and kinetics, as well as the role of binding in induced folding in biomolecular recognition. Our results provide a novel way to understand flexible biomolecular binding, especially IDPs' binding-folding dynamics and therefore have a wide application in theory and experiments.

## Materials and Methods

Using coarse grained structure based model [99]–[102], we quantified the intrinsic energy landscapes by exploring density of states, which is a distribution in micro-canonical ensemble. However, molecular simulations are usually performed at constant temperature, or volume, or pressure, corresponding to canonical ensemble. Through the thermodynamic relationship between micro-canonical and canonical ensemble: 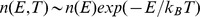, we can obtain the density of states and then quantified the intrinsic energy landscapes. Our simulations were performed using Langevin equation encoded in Gromacs 4.0.5 [103]. Topology files for Gromacs were generated using the SMOG@ctbp webserver (http://smog-server.org) [100]. Reduced unit were used throughout our work. Density of states was calculated from thermodynamic simulations, which were preformed using Replica Exchanged Molecular Dynamics (REMD) [104] with 48 parallel temperatures ranging from 0.2 to 2.2. Each replica ran with 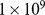 steps and attempted to exchange with its neighbor replicas at every 20000 steps. The average acceptance ratio for each thermodynamic simulation is found to be 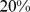 to 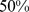, leading to sufficient data sampling. After that, we collected the data and transformed the distributions into micro-canonical ensemble using Weighted Histogram Analysis Method (WHAM) algorithm [105]. In order to calculate binding rate, we performed additional kinetic simulations with 200 independent constant temperature trajectories at each temperature, started from varying dissociative chains. First passage time (FPT) was collected for each trajectory and finally mean first passage time (MFPT) was calculated as binding time.

For binding, rigid binding is realized by freezing the monomers of the investigated homodimers and therefore binding actually happens between two identical rigid chains. In this way, the monomeric folding and configurational plasticity are removed and the dynamics is only related to interfacial binding. To be consistent, for rigid binding, we used the same interfacial binding interactions, namely the same interfacial binding contact map, as used in flexible binding, which has been investigated in our previous work [57]. Rigid binding, different with flexible binding, which is strongly coupled with the monomeric folding during the recognition process, can be regarded as independent binding. Two identical chains of each homodimers were placed at the center of a 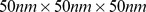 cubic box. To enhance the sampling of binding events, a strong harmonic potential was added if the distance between the center of mass of the two chains is farther than 6 nm, corresponding to an effective protein concentration 3.6 mM. By investigating the native structure of the five homodimers, we found that the distance of the center of mass of the two subunits in the homodimers are all smaller than 2.8 nm, so this confinement is supposed to have no effect on the native states. Both in simulations and experiments, the confinement in a moderate strength imposed on protein binding has been found to be a passive, largely entropic role on unbound states to enhance the binding rate and stability [106]. However, the binding mechanism is found to be not changed qualitatively [65], [107]. For folding, the isolated monomeric folding is realized by folding the monomers without dimeric environment and therefore can be regarded as independent folding. To be consistent, for isolated folding, we used the same monomeric folding interactions, namely the same monomeric contact map, as used in dimeric folding, which has been investigated in our previous work [57]. Simulation and calculation details can be found in Text S1 and our previous work [57], [62].

For quantifying the combined binding-folding energy landscapes, we used the conclusions in our theoretical work [63]. In practice, we calculated the combined density of states from the three independent density of states by:
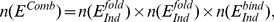
so 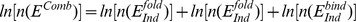
and similarly
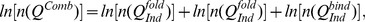
where 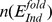, 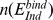, 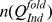 and 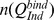 are the density of states of independent folding and binding in energy and configuration space. Notice that the homodimers are comprised of two identical chains, the folding properties of the two monomers are expected to be the same. The topography quantities, such as energy gap 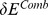, roughness 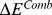 and entropy 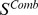 of the combined energy landscapes can be deduced from the corresponding quantities from independent binding and folding energy landscapes.
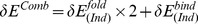





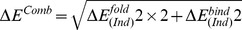





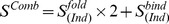
where 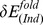, 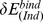, 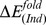, 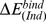, 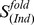 and 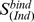 are the energy roughness, gap and entropy of independent folding and binding energy landscapes. Finally, we can obtain the combined energy landscape topography measure 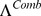 as:
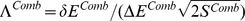



## Supporting Information

Text S1 Models and simulation details, structural characteristics and additional results.
(PDF)
