# Supplementary material for: Specificity and Affinity Quantification of Flexible Recognition from Underlying Energy Landscape Topography
Source: PLoS Comput Biol. 2014 Aug 21;10(8):e1003782. doi: 10.1371/journal.pcbi.1003782 (PMC4140643; doi:10.1371/journal.pcbi.1003782)
Supplement: Text S1 — Models and simulation details, structural characteristics and additional results. (PDF) [file pcbi.1003782.s001.pdf]

Text S1

1 Models and simulation details

Based on the energy landscape theory [1–5], structure based model, which only takes into account the native interactions, has been widely used for investigating the protein folding/binding dynamics and can successfully reproduce experimental measurements through free energy profiles and Φ value analysis [6–9]. In our coarse grained model, each animo acid is represented by a single bead located at its C^{α} atom position. Native interactions participate into the energy balance by same weight without consideration of the physicochemical property of different residues. A typical C^{α} Hamiltonian function at configuration Γ is given by:

$$
\begin{aligned}
H(\Gamma,\Gamma_0)_{SBM} = \quad & \sum_{bonds} K_r(r-r_0)^2 + \sum_{angles} K_\theta(\theta-\theta_0)^2 \\
& + \sum_{dihedral} K_\phi^{(n)}[1-cos(n\times(\phi-\phi_0))] \\
& + \sum_{i<j-3}^{native} \epsilon(i,j)[5(\frac{\sigma_{ij}}{r_{ij}})^{12} - 6(\frac{\sigma_{ij}}{r_{ij}})^{10}] \\
& + \sum_{i<j-3}^{non-native} \epsilon_2(i,j)(\frac{\sigma_{NC}}{r_{ij}})^{12}
\end{aligned}
$$

where r, θ, ϕ are virtual bond length, bond angle and dihedral, representing bond stretching, angle bending and torsional interactions, respectively. The parameters with subscript zero represents the corresponding values adopted in the native binding structure, Γ_0. Non-bonded interactions are considered only when two C^{α} atoms i and j are separated sequentially by at least three residues on a chain or when they belong to different chains and are subdivided into native interactions and non-native interactions. For native interactions, σ_{ij} is the distance between C^{α} atoms forming native contacts. For non-native contacts, $\sigma_{NC} = 4\mathring{A}$, represents the excluded volume repulsion. The native contact map is built by the Contacts of Structural Units (CSU) software [10]. Reduced units are used for all calculations with $\epsilon = 1.0$, so $K_r = 100.0$, $K_\theta = 20.0$, $K_\phi^{(1)} = 1.0$, $K_\phi^{(3)} = 0.5$ and $\epsilon_2 = 1.0$.

All the thermodynamic and kinetic simulations were performed with Gromacs 4.0.5 [11]. We generated and simulated the system using a standard step proposed by SMOG@ctbp webserver (http://smog-server.org) [12]. The coarse grained molecular dynamics (CGMD) simulations used Langevin equation with constant friction coefficient $\gamma = 1.0$. The MD time step was set to 0.5 fs and the trajectories were saved at every 1 ps. Thermodynamic simulations were preformed using Replica Exchanged Molecular Dynamics (REMD) [13] with 48 parallel temperatures ranging from 0.2 to 2.2. Each replica attempted to exchange with its neighbor replicas at every 20000 MD steps (10 ps). The average acceptance ratio for each thermodynamic simulation were found to be 20% to 50%, leading to sufficient data sampling. Kinetic simulations were performed by 200 independent constant temperature trajectories at each temperature, started from varying dissociative chains. First passage time (FPT) is collected and the mean first passage time (MFPT) is regarded as the binding time. In our CGMD, temperature is the only factor to mimic the different environments. We use the temperature T_χ^{bind}, at which the bound states occupy 80% population, as the kinetic temperature. The kinetic temperature T_χ^{bind} is supposed to be lower than the binding transition temperature, and therefore mimics the physiological conditions to accelerate the biomolecular recognition, compared with that at the binding transition temperatures.

The three quantities of topography of energy landscapes: energy gap δE, entropy S and energy roughness ΔE can be calculated from the underlying density of states, which is obtained by thermodynamic

simulations. The energy gap, which measures the slope of the funnel [14,15], is calculated by the energy gap between native state and average of non-native states: $\delta E = |E_{native} - < E_{non-native} > |$. The entropy, which measures the size of the funnel, can be calculated by the logarithm of the density of states at non-native states. The energy roughness, which measures the bumpiness of the funnel, can be calculated by the expression: $\Delta E = \sqrt{2S}T_g$, where T_g is the glassy trapping temperature and can be calculated by the slope of logarithm of density of states of non-native states at ground states by $1/T = \partial S/\partial E$ [16,17]. With these three quantities, we can quantify the energy landscape topography measure $\Lambda = \delta E/(\Delta E\sqrt{2S})$. Further details can be found there [16,17].

2 Structural characteristics

Table S1. Structural and topological properties of the five 3-state homodimers

**Protein**	PDB code	N [a]	Monomeric NC [b]	Interfacial NC	Total NC	Q_{Coupled}
Lambda Cro repressor	1cop	132	118(115)	58	291	0.18
Lambda repressor	1lmb	174	191(190)	49	430	0.17
LFB1 transcription factor	1lfb	154	147(147)	50	344	0.16
Cytochrome b5 type B	3mus	170	177(173)	41	391	0.12
Superoxide dismutase	1xso	300	438(435)	60	933	0.09

[a] N is the number of residues of the homodimer.
[b] NC is the native contact number. There are two values for monomeric NC, each for one chain.

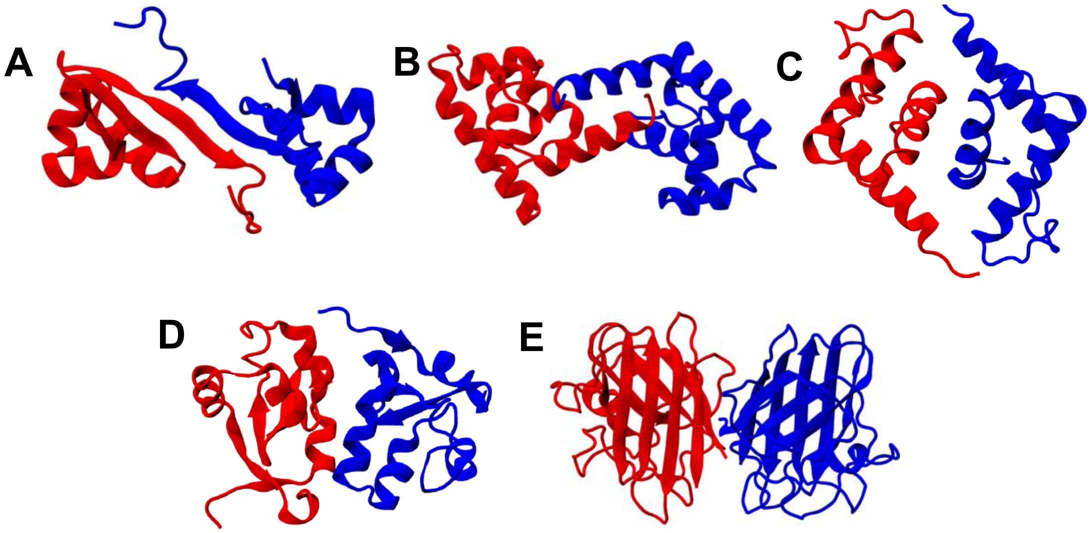

Figure S1. The native structure of the five homodimers are shown in cartoon representation. (A) Lambda Cro repressor (PDB: 1cop). Lambda Cro repressor is a gene regulating protein. With binding to DNA, it controls the genetic switch [18]. (B) Lambda repressor (PDB: 1lmb). Lambda repressor is a transcriptional regulator. With binding to adjacent sits on DNA, it maintains the life cycle [19]. (C) LFB1 transcription factor (PDB: 1lfb). LFB1 transcription factor is a transcriptional regulator. It is a regulator of liver-specific gene expression in mammals [20]. (D) Cytochrome b5 type B (PDB: 3mus). Cytochrome b5 type B is a monomer which functions as an electron carrier [21]. The homodimer used in our simulations comes from crystal packing. (E) Superoxide dismutase (PDB: 1xso). Superoxide dismutase is enzyme that catalyzes the dismutation of superoxide into oxygen and hydrogen peroxide [22]. The two chains of each homodimer are shown in different colors. The structures are created using the package Visual Molecular Dynamics (VMD) [23].

3 Results

3.1 Rigid and flexible binding

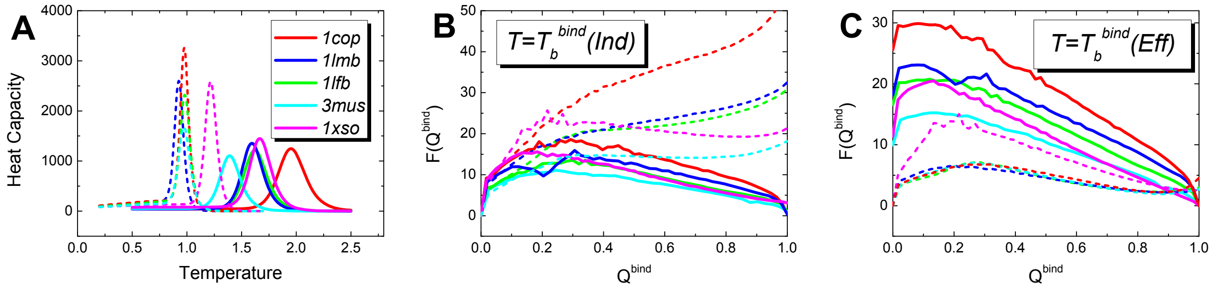

Figure S2. The binding affinity (stability) for rigid (independent) and flexible (effective) binding shown in (A) heat capacity curves and (B, C) free energy landscapes. The solid and corresponding dotted lines represent rigid and flexible binding, respectively. "Ind" and "Eff" are the abbreviations for "Independent" and "Effective" binding, respectively.

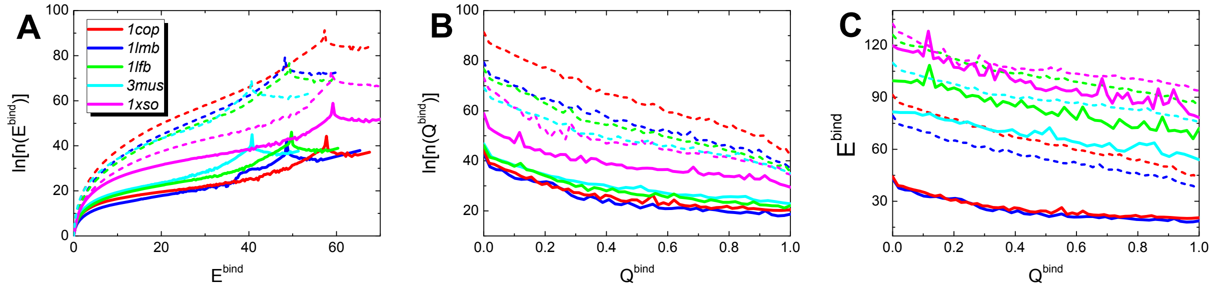

Figure S3. The rigid and flexible binding energy landscapes. Logarithm of density of states are plotted as a function of (A) interfacial binding energy E^{bind} and (B) fraction of native interfacial binding contacts Q^{bind}. (C) The interfacial binding energy E^{bind} is plotted as a function of fraction of native interfacial binding contacts Q^{bind}. The solid and corresponding dotted lines represent rigid and flexible binding, respectively. The lowest energy (energy of native structure) is set to 0 for a better visualization. Energy is in reduced unit.

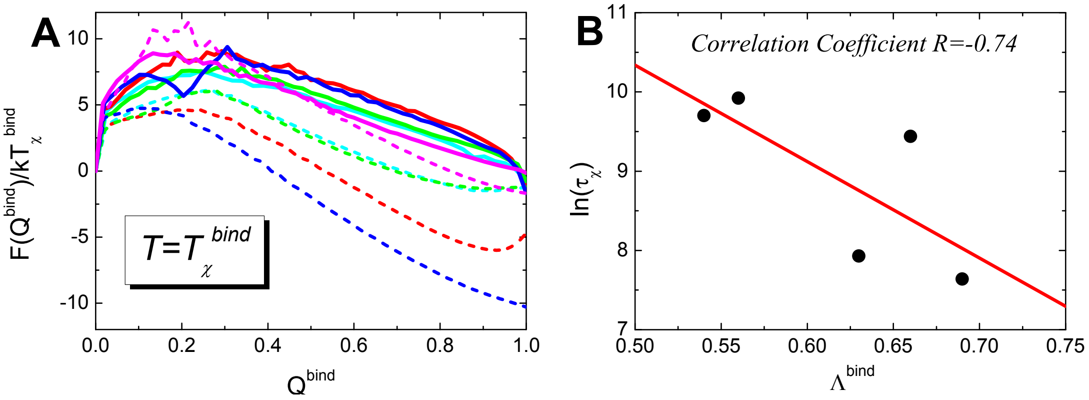

Figure S4. (A) The differences of free energy profiles between rigid and flexible binding at kinetic temperature T_χ. Free energy is in the unit of kT_χ. (B) The relationship between the association time $ln(\tau_\chi)$ and topography measure Λ^{bind} for rigid binding. The red line is linear fit and the correlation coefficient is -0.74. The color representation in (A) is same with that in Figure S2 and Figure S3.

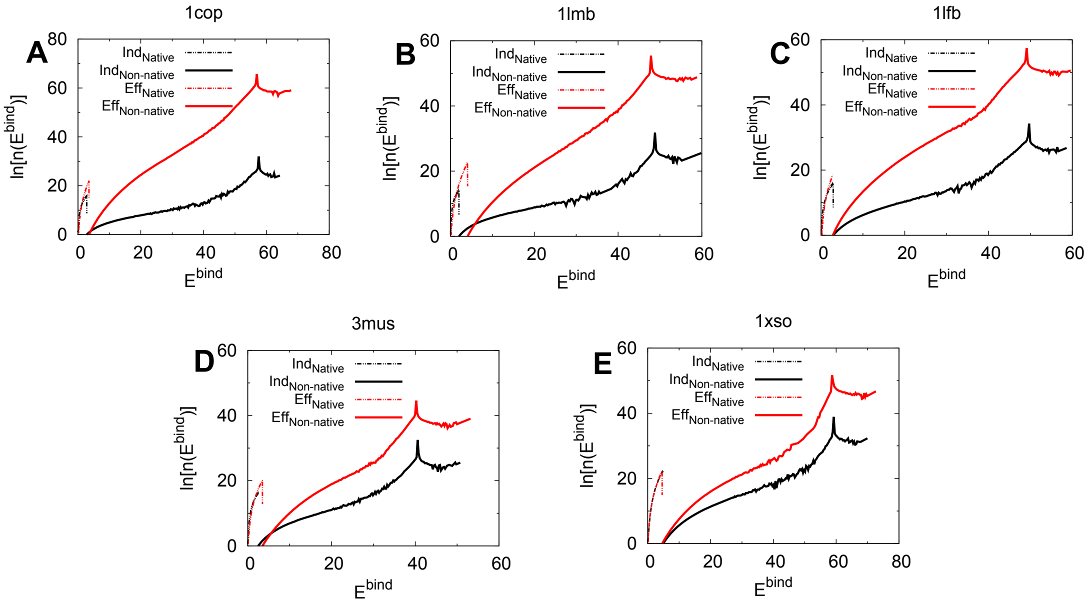

Figure S5. The rigid and flexible binding energy landscapes. Logarithm of density of states of native and non-native states are plotted as a function of interfacial binding energy E^{bind}. The lowest energy (energy of native structure) is set to 0 for a better visualization. Energy is in reduced unit.

3.2 Folding with and without binding

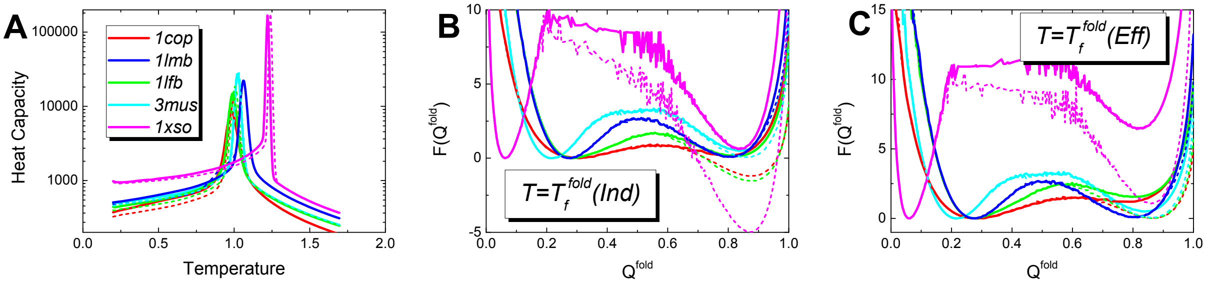

Figure S6. The folding stability for folding with and without interfacial binding shown in (A) heat capacity curves and (B, C) free energy landscapes. The solid and corresponding dotted lines represent isolated (independent) and dimeric (effective) folding respectively. Free energy landscapes are plotted at the (B) isolated and (C) dimeric folding transition temperatures, which are calculated from the peaks of heat capacity curves for folding, respectively. Free energy is in reduced unit. Q^{fold} is the fraction of native monomeric folding contacts. "Ind" and "Eff" are the abbreviations for "Independent" and "Effective" folding, respectively.

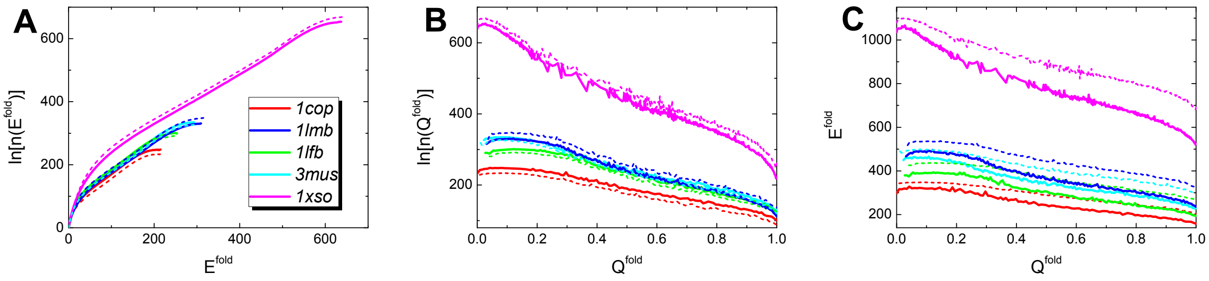

Figure S7. The folding energy landscapes with and without interfacial binding. Logarithm of density of states are plotted as a function of (A) monomeric folding energy E^{fold} and (B) fraction of native monomeric folding contacts Q^{fold}. (C) The monomeric folding energy E^{fold} is plotted as a function of fraction of native monomeric folding contacts Q^{fold}. The solid and corresponding dotted lines represent isolated and dimeric folding respectively. The lowest energy (energy of native structure) is set to 0 for a better visualization. Energy is in reduced unit.

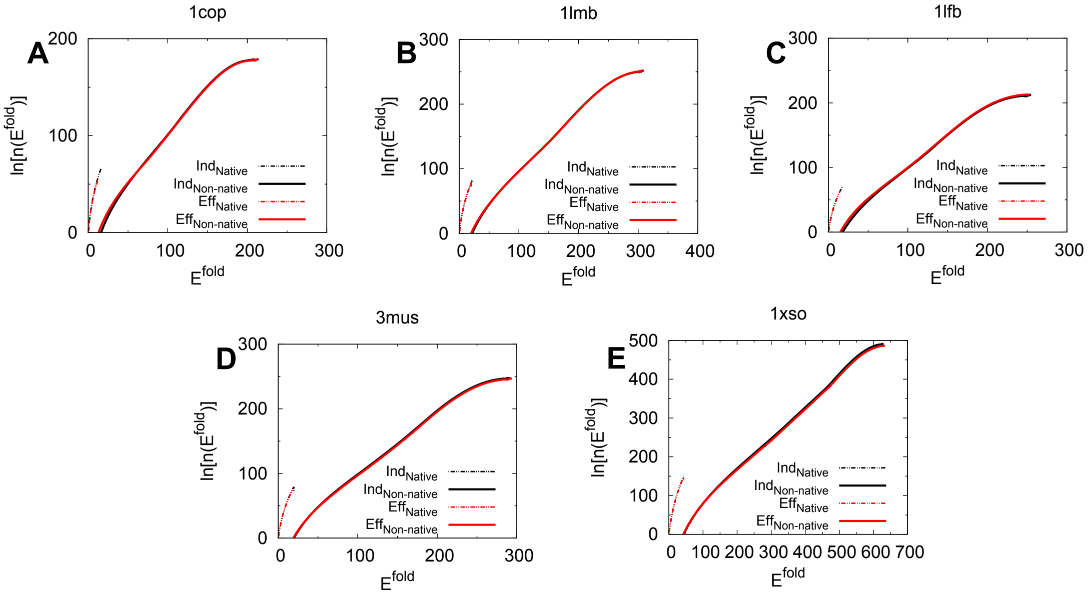

Figure S8. The folding energy landscapes with and without interfacial binding. Logarithm of density of states of native and non-native states are plotted as a function of monomeric folding energy E^{fold}. The lowest energy (energy of native structure) is set to 0 for a better visualization. Energy is in reduced unit.

3.3 The combined and the whole global binding-folding energy landscapes

Table S2. Quantified the combined and the whole global binding-folding energy landscapes.

**Proteins**	**1cop**		**1lmb**		**1lfb**		**3mus**		**1xso**	
	Comb	Glob	Comb	Glob	Comb	Glob	Comb	Glob	Comb	Glob
δE^{whole} [a]	469.46	454.59	656.47	632.80	546.62	520.92	611.27	594.26	1311.55	1291.85
ΔE^{whole}	17.20	13.86	18.98	17.18	16.44	14.80	16.91	16.03	26.13	26.10
S^{whole} [b]	420.46	417.14	563.86	546.00	487.21	475.63	556.19	535.14	1049.45	1012.84
T_b^{whole} [c]	0.99	0.99	1.06	1.06	1.00	1.00	1.02	1.02	1.24	1.24
T_g^{whole} [d]	0.60	0.48	0.57	0.52	0.53	0.48	0.52	0.49	0.58	0.58
T_b^{whole}/T_g^{whole}	1.65	2.07	1.86	2.03	1.89	2.09	1.96	2.08	2.14	2.14
Λ^{whole}	0.94	1.13	1.03	1.11	1.07	1.14	1.08	1.13	1.09	1.10
$Q_{coupled}$	0.18		0.17		0.16		0.12		0.09	

[a] The superscript "whole" can be "Comb" and "Glob", corresponding to the combined and the whole global energy landscapes, respectively.
[b] S^{whole} is the configuration entropy of the non-native states. For the combined energy landscapes, S^{Comb} is equal to the logarithm of density of states of the combined non-native states.
[c] T_b is the binding transition temperature, obtained from heat capacity of binding-folding and can be explicitly measured in experiments.
[d] The glassy trapping temperature of the combined binding-folding energy landscape is expressed by: $T_g^{Comb} = \sqrt{(\Delta E_{Ind}^{fold^2} \times 2 + \Delta E_{Ind}^{bind^2})/2S^{Comb}}$.

References

1. Bryngelson JD, Wolynes PG (1987) Spin-glasses and the statistical-mechanics of protein folding. Proc Natl Acad Sci USA 84: 7524-7528.

2. Bryngelson JD, Wolynes PG (1989) Intermediates and barrier crossing in a random energy-model (with applications to protein folding). J Phys Chem 93: 6902-6915.

3. Leopold PE, Montal M, Onuchic JN (1992) Protein folding funnels - a kinetic approach to the sequence structure relationship. Proc Natl Acad Sci USA 89: 8721-8725.

4. Dill KA, Chan HS (1997) From levinthal to pathways to funnels. Nat Struct Biol 4: 10-19.

5. Wolynes PG, Onuchic JN, Thirumalai D (1995) Navigating the folding routes. Science 267: 1619-1620.

6. Clementi C, Nymeyer H, Onuchic JN (2000) Topological and energetic factors: What determines the structural details of the transition state ensemble and ”en-route” intermediates for protein folding? an investigation for small globular proteins. J Mol Biol 298: 937-953.

7. Levy Y, Wolynes PG, Onuchic JN (2004) Protein topology determines binding mechanism. Proc Natl Acad Sci USA 101: 511-516.

8. Levy Y, Cho SS, Onuchic JN, Wolynes PG (2005) A survey of flexible protein binding mechanisms and their transition states using native topology based energy landscapes. J Mol Biol 346: 1121-1145.

9. Levy Y, Onuchic JN (2006) Mechanisms of protein assembly: Lessons from minimalist models. Acc Chem Res 39: 135-142.

10. Sobolev V, Sorokine A, Prilusky J, Abola EE, Edelman M (1999) Automated analysis of interatomic contacts in proteins. Bioinformatics 15: 327-332.

11. Hess B, Kutzner C, van der Spoel D, Lindahl E (2008) Gromacs 4: Algorithms for highly efficient, load-balanced, and scalable molecular simulation. J Chem Theory Comput 4: 435-447.

12. Noel JK, Whitford PC, Sanbonmatsu KY, Onuchic JN (2010) Smog@ctbp: simplified deployment of structure-based models in gromacs. Nucleic Acids Res 38: W657-W661.

13. Okamoto Y, Sugita Y (1999) Replica-exchange molecular dynamics method for protein folding. Chem Phys Lett 314: 141-151.

14. Plotkin SS, Wang J, Wolynes PG (1997) Statistical mechanics of a correlated energy landscape model for protein folding funnels. J Chem Phys 106: 2932-2948.

15. Plotkin SS, Wang J, Wolynes PG (1997) Statistical mechanics of correlated energy landscape models for random heteropolymers and proteins. Physica D 107: 322-325.

16. Wang J, Oliveira RJ, Chu X, Whitford PC, Chahine J, et al. (2012) Topography of funneled landscapes determines the thermodynamics and kinetics of protein folding. Proc Natl Acad Sci USA 109: 15763-8.

17. Chu X, Gan L, Wang E, Wang J (2013) Quantifying the topography of the intrinsic energy landscape of flexible biomolecular recognition. Proc Natl Acad Sci USA 110: E2342-E2351.

18. Matsuo H, Shirakawa M, Kyogoku Y (1995) Three-dimensional dimer structure of the λ-cro repressor in solution as determined by heteronuclear multidimensional nmr. J Mol Biol 254: 668–680.

19. Beamer LJ, Pabo CO (1992) Refined 1.8 å crystal structure of the λ repressor-operator complex. J Mol Biol 227: 177–196.

20. Ceska T, Lamers M, Monaci P, Nicosia A, Cortese R, et al. (1993) The x-ray structure of an atypical homeodomain present in the rat liver transcription factor lfb1/hnf1 and implications for dna binding. EMBO J 12: 1805.

21. Parthasarathy S, Altuve A, Terzyan S, Zhang X, Kuczera K, et al. (2011) Accommodating a nonconservative internal mutation by water-mediated hydrogen bonding between β-sheet strands: A comparison of human and rat type b (mitochondrial) cytochrome b 5. Biochemistry 50: 5544–5554.

22. Djinovic Carugo K, Battistoni A, Carri M, Polticelli F, Desideri A, et al. (1996) Three-dimensional structure of xenopus laevis cu, zn superoxide dismutase b determined by x-ray crystallography at 1.5 a resolution. Acta Crystallogr D 52: 176–188.

23. Humphrey W, Dalke A, Schulten K (1996) Vmd: Visual molecular dynamics. J Mol Graph 14: 33-38.
